# Perspectives on Ligand/Protein Binding Kinetics Simulations: Force Fields, Machine Learning, Sampling, and User-Friendliness

**DOI:** 10.1021/acs.jctc.3c00641

**Published:** 2023-09-01

**Authors:** Paolo Conflitti, Stefano Raniolo, Vittorio Limongelli

**Affiliations:** † Faculty of Biomedical Sciences, Euler Institute, Universitá della Svizzera italiana (USI), 6900 Lugano, Switzerland; ‡ Department of Pharmacy, University of Naples “Federico II”, 80131 Naples, Italy

## Abstract

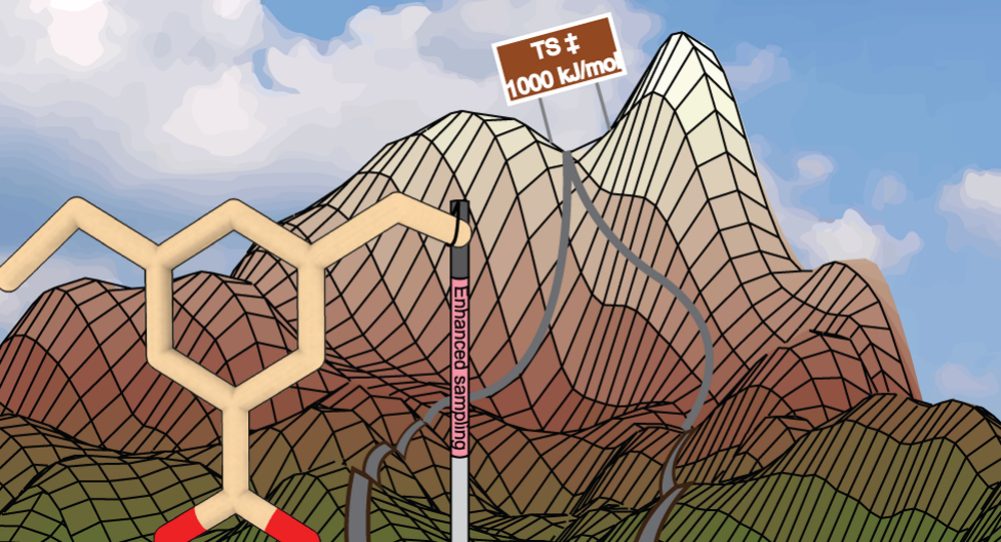

Computational techniques applied to drug discovery have gained considerable popularity for their ability to filter potentially active drugs from inactive ones, reducing the time scale and costs of preclinical investigations. The main focus of these studies has historically been the search for compounds endowed with high affinity for a specific molecular target to ensure the formation of stable and long-lasting complexes. Recent evidence has also correlated the in vivo drug efficacy with its binding kinetics, thus opening new fascinating scenarios for ligand/protein binding kinetic simulations in drug discovery. The present article examines the state of the art in the field, providing a brief summary of the most popular and advanced ligand/protein binding kinetics techniques and evaluating their current limitations and the potential solutions to reach more accurate kinetic models. Particular emphasis is put on the need for a paradigm change in the present methodologies toward ligand and protein parametrization, the force field problem, characterization of the transition states, the sampling issue, and algorithms’ performance, user-friendliness, and data openness.

## 1 Introduction

**Figure 1:**
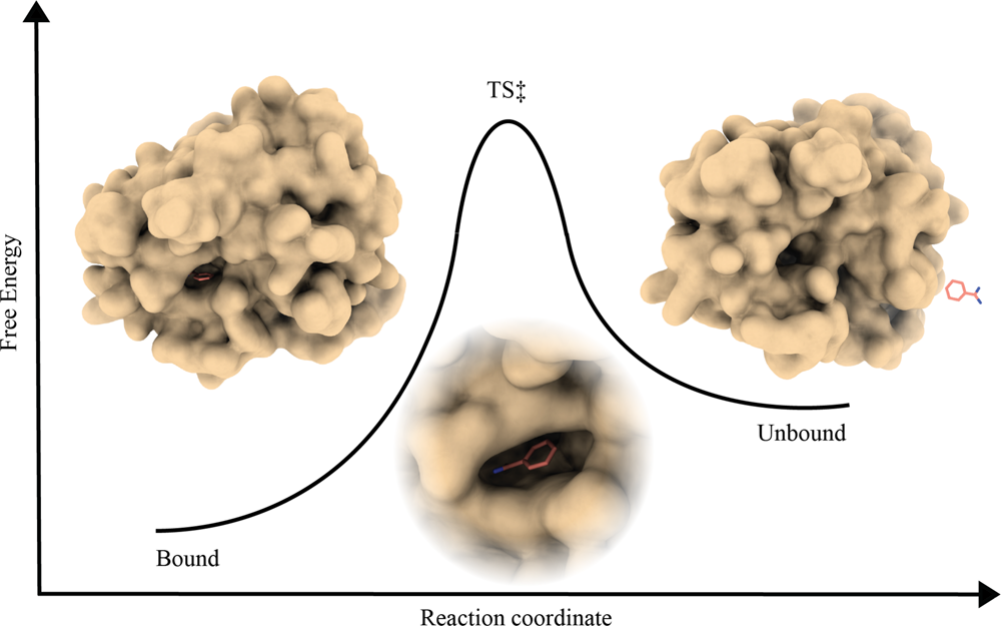
Artistic representation of the free-energy profile of LPB with bound (Bound), transition state (TS) and unbound state (Unbound). Target protein and ligand are shown as the surface and licorice, respectively.

The pharmacological properties of a drug are typically defined as the pharmacokinetic and pharmacodynamic properties. While pharmacokinetics regards the body effect on the drug defining its absorption, distribution, metabolism, excretion, and toxicity (i.e., ADMET), pharmacodynamics deals with the drug’s effect on our body, which can be reasonably rationalized by its mechanism of action and the elucidation at atomistic scale of the drug binding interaction with its molecular target. The latter is of paramount relevance to guide drug discovery and is the topic of the present article. Despite the recent methodological advances, drug discovery remains a daunting task with poorly performing predictive models of in vivo drug efficacy,^1,2^ dramatic time scale, and exorbitant costs (10–15 years and 2.6 billion US dollars on average to develop a new medication).^3,4^ Considering also the low success rate of a drug to pass clinical trials (below 10%),^5^ it is apparent that there is a tremendous need for techniques capable of increasing the probability that a ligand obtained from basic research could become a drug and at the same time reducing the costs of the research.^6−8^ This need is even more urgent since December 2022 when the Food and Drug Administration (FDA) took the historic decision to replace the word “animal” with “nonclinical tests” in the law governing the agency’s drug assessments, paving the way to nonanimal alternative methods, such as organoids, organs-on-a-chip and in silico modeling (Food and Drug Administration Modernization Act 2.0).^9^ In this context, structure-based drug discovery (SBDD) will play an ever more prominent role. Traditionally, SBDD relies on drug/target binding studies (hereafter ligand/protein binding, LPB) focusing on ligand affinity to the target, expressed as ligand binding free energy Δ*G*, binding/dissociation constant K_*b*_/K_*d*_, or half maximal effective/inhibitory concentration EC50/IC50. However, this is an oversimplified representation of LPB, which is by far a more complex molecular process where the ligand can reach the final binding mode by passing through metastable states (alternative binding modes) and crossing even high energy barriers (Figure 1).

In other words, LPB can be seen as a combination of thermodynamic and kinetic problems, the first defining the ligand binding affinity to its target by estimating the ratio between the ligand concentration in the bound and unbound states and the second characterizing the ligand residence time in the target by computing the rate of ligand (un)binding. The thermodynamics and kinetics of LPB are properties of the system and are quantified as constants related by the following eq 1:
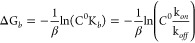
1where ΔG is the free-energy difference between the ligand-bound and unbound state, K_*b*_ is the binding constant (K_*d*_ is the inverse of K_*b*_), β is the inverse of the product of the Boltzmann constant and temperature, C^0^ is the standard concentration of 1 M, also expressed as 1/1660 Å^–3^, and k_*on*_ and k_*off*_ are the two kinetics constants for the binding and unbinding processes, respectively.

Noteworthily, recent evidence has correlated ligand binding rates (kinetics) more than ligand binding affinity (thermodynamics) to in vivo drug efficacy.^10−16^ In addition, ligand binding kinetics have also been related to the type of ligand activity, agonist or antagonist, letting glimpse the potential impact of including ligand binding kinetics in drug discovery.^17^ However, drug discovery studies are still rooted in binding affinity predictive models, and no drug has been developed based on ligand binding rate prediction so far. The reasons for this are both historical and methodological. While plenty of binding free-energy methods have been developed in the last four decades, ligand-binding kinetic models have only appeared in the last two.^18,19^ Furthermore, the prediction of ligand binding rates requires the characterization of LPB transition states (TSs), which are high-energy, transient (short-lived) states that are elusive to standard structural biology methodologies. Encouraging results come from time-resolved X-ray crystallography and cryogenic electron microscopy, which provide an augmented spatial and temporal resolution of nonequilibrium macromolecule states.^20,21^ Similarly, nuclear magnetic resonance (NMR),^22^ surface plasmon resonance,^23,24^ and other techniques (see Bernetti et al. 2019 for review)^14^ have made significant progress in providing a dynamic description of LPB for kinetic predictions. However, all these techniques are time-demanding and costly, and they hardly provide mechanistic structural details useful to guide drug design.^25^ To this end, computational approaches are valuable, since they can sample TSs and obtain transition rates from one energy minimum to another. Having the atomistic structure of TS at the saddle point and the ligand binding mode provides a unique, comprehensive description of LPB that might impact the quality and success of structure–activity relationship (SAR) studies and drug discovery in general. However, so far molecular simulations are used to reproduce experimental kinetic data, with only few examples where calculations are presented together with or followed by experimental validation.^26−29^ This is mainly due to two limiting factors that are the time required to complete a kinetic study, in terms of both simulation and real time for analysis, and the accuracy of the calculations that largely rely on system-dependent simulation settings, which hamper a routine and automated use of such techniques in drug discovery. We discuss such aspects in the following paragraphs, describing the state-of-the-art techniques employed in LPB kinetics studies and their limits as predictive tools. We further delineate the future directions of in silico approaches in order to have a significant impact on drug discovery.

## 2 State of the Art

Many approaches aimed at studying LPB kinetics have been developed so far. They can be grouped into unbiased and biased MD-based methods.

The first category comprises techniques that focus on sampling the transition between metastable states by massively parallelizing the simulation, allowing direct calculation of the kinetic properties. Milestoning with MD or Brownian Dynamics (BD) simulations,^30−33^ its notable variation called Markovian Milestoning with Voronoi Tessellations (MMVT),^34^ Weighted Ensemble Methods (WEM and Markovian-WEM),^35−39^ and Adaptive Multilevel Splitting (AMS)^40,41^ are four examples of this kind of approach. Their key concept is discretizing the configuration space using various descriptors (e.g., grids, distances, native contacts). Multiple simulations are then spawned or killed semiindependently to ensure a statistically meaningful exploration of the transition pathways in a reasonable time. Although these techniques are all based on unbiased approaches, i.e., the single simulations are not affected by external bias, the sampling algorithm may follow a nonequilibrium strategy. In fact, in sampling at equilibrium, the system explores the forward and backward transitions of energy barriers without altering the probability distribution. On the other hand, in nonequilibrium samplig the reconstruction of the equilibrium ensemble is possible by optimizing the number of simulations performed, their distribution, or relative weights, so as to properly sample the rare event and promote the transition in a specific direction. According to this definition, Milestoning and MMVT are equilibrium approaches, whereas WE and AMS are not.

At variance with unbiased simulations, in biased techniques, user-defined degrees of freedom of the system are accelerated by adding an external potential or force, which might be used to compute the correct (unbiased) estimate of the energetic barriers crossed during the simulation. Umbrella Sampling (US),^42^ Steered MD (stMD),^43^ Targeted MD (TMD) and Dissipation-Corrected Targeted MD (dcTMD),^44−46^ Smoothed MD (SMD),^47,48^ Adiabatic-bias MD (AbMD),^49,50^ Metadynamics MD (MetaD) (i.e., infrequent Metadynamics, frequency-adaptive metadynamics),^51−54^ On-the-fly Probability-Enhanced Sampling (OPES, and its flooding variant OPES_*f*_),^55−57^ Gaussian Accelerated MD (GaMD) and its variants (Pep-GaMD, LiGaMD),^58−60^ and τ-random acceleration MD (τRAMD)^61^ are notable examples of this family of out-of-equilibrium approaches. Among these, we note that in infrequent MetaD and OPES_*f*_, while the sampling of the basins is accelerated, no bias should be applied to the system during the transition between them. These techniques can be further classified according to how the external potential, or force, is applied to the system and how the kinetic properties are estimated. US, MetaD, AbMD, stMD, TMD, and dcTMD require the definition of specific Collective Variables (CVs), which should describe the slowest degrees of freedom of the process investigated.^42,43,45,46,50,51,62^ On the other hand, in GaMD, harmonic boost potentials are directly applied to the potential energy of the system.^60^ Similarly, in SMD, the potential energy function is scaled back by a user-defined factor.^47^ In τRAMD, randomly oriented forces are applied to the ligand to accelerate its unbinding.^61^ Some of these methods compute the kinetic data by estimating the energetic barriers for the binding or unbinding events using Kramers’ rate theory or the Eyring equation. This is the case of stMD, dcTMD, and GaMD.^43,44,60^ This approach could be also applied to MetaD and OPES. Using the latter techniques, it is also possible to directly estimate the accelerated ligand residence time, which is then rescaled to the correct (unbiased) one using the bias deposited during the simulation.^53,56^ The association (binding) rate might be finally derived from the dissociation constant and the ligand residence time.^53^ Finally, SMD and τRAMD provide a computational residence time that can be correlated with the available experimental data, whereas abMD allows for estimating an energetic score that can be correlated to the experimental residence time.

In between biased and unbiased computational methods, we find Markov State Models (MSMs).^63−65^ MSMs do not have a defined simulation protocol but rather represent an a posteriori analysis strategy that can be applied to any set of biased or unbiased calculations.^53,66−68^ The only requirements are that these simulations should be capable of discretizing the phase space into a number of different, energetically relevant states, thus allowing the computation of the transition matrix with the exchange probability among these states.

We refer the reader to ref (19) for a review and deeper discussion of these methods. In the following sections, we focus on the limitations and advantages of these and other computational strategies, providing insights into much-needed improvements required to allow a breakthrough in the field. They can be summarized in three points:

- the force fields issue;
- the sampling issue;
- performance, user-friendliness, and data openness.

## 3 Force Fields

**Figure 2:**
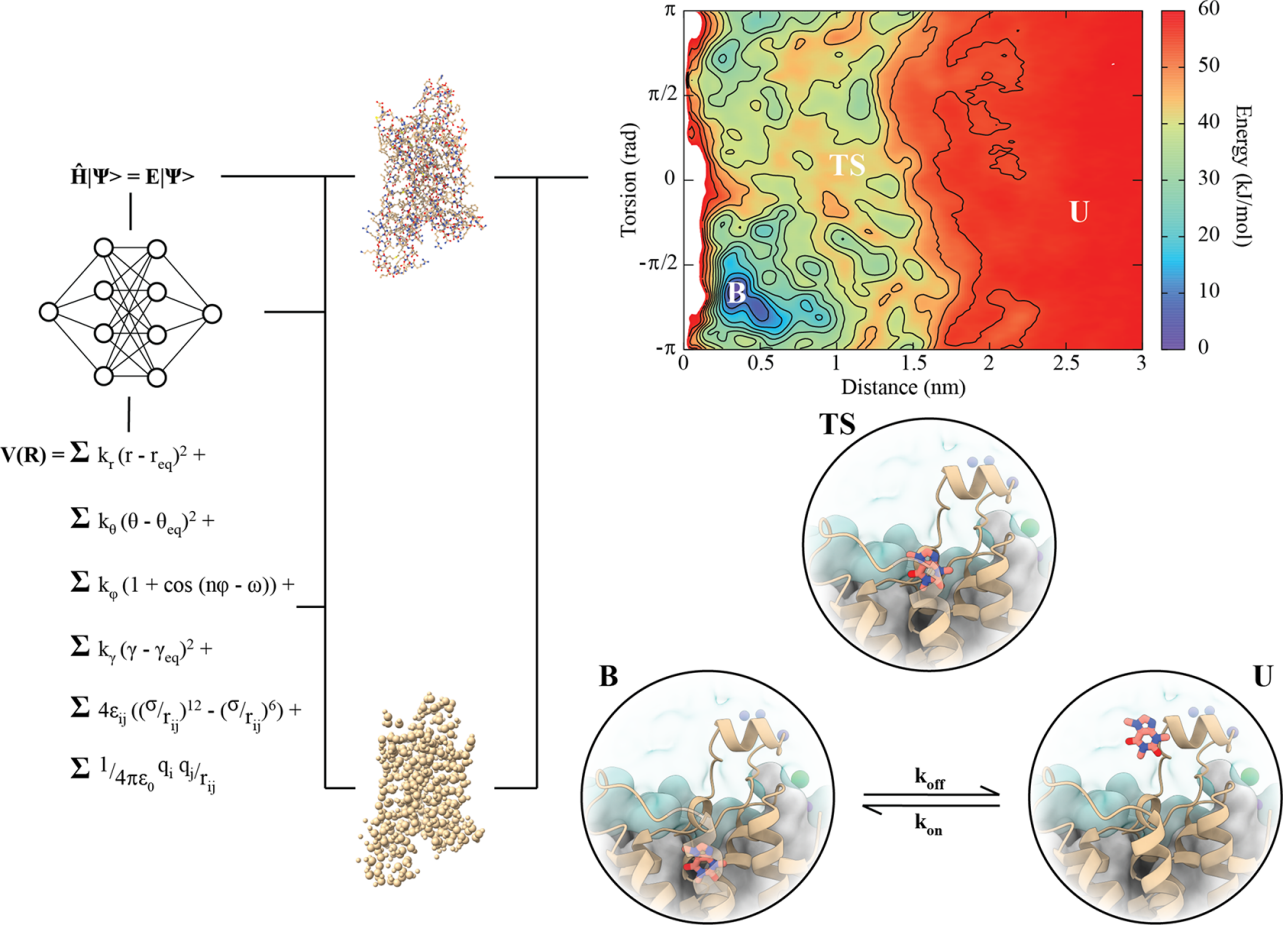
Pipeline of LPB kinetics calculations for a sample system (i.e., adenosine GPCR A2A in complex with caffeine). The LPB can be parametrized using classic or ML-based FFs and then simulated employing one of the computational techniques described in the text. The output is the identification of the possible energy-minima LPB complexes (B and U), the transition state (TS), and rates between them (k_*on*_ and k_*off*_).

Force Fields (FFs) are a set of parameters through which interatomic forces are computed, allowing the system to evolve during an atomistic simulation, be it MD or Monte Carlo. As such, FFs should describe the physics underlying the simulated phenomenon, and their accuracy is crucial for the predictive power of simulations (Figure 2).

Over the years, continuous efforts have been paid in the development of reliable FFs for biological macromolecules (e.g., Amber, CHARMM, OPLS)^69−74^ and small organic molecules (e.g., GAFF, CGenFF, OPLS, OpenFF, SMIRNOFF99Frosst).^69,73−77^ Typically, atomistic FFs employ classical physics equations to describe interatomic interactions with fixed, point-charge models, defined without considering the surrounding chemical environment. The parameters of these equations are derived from experimental or quantum mechanical (QM) calculations data, often using models with a finite number of atoms. These are then adapted into more generalized terms to serve a wider variety of chemical entities.^69−74^ Despite the intrinsic limitations of these models justified by historical and practical reasons (FFs were developed using significantly lower computational capabilities compared to today), they have served very well the purpose of advancing our understanding of biophysical processes. But are classical FFs accurate enough for modern research in the age of GPU accelerators and machine learning (ML) techniques? The answer is highly dependent on the problem of interest. We can start by saying that in the case of LPB kinetics traditional FFs are not accurate enough.

An accurate estimate of kinetic rates requires a proper identification of TSs.^10^ However, subpar parameters might overly stabilize specific ligand (and protein) conformations, thus altering the identification and energetic evaluation of metastable and transient states.^78,79^ It has been shown that inadequacies in the van der Waals and electrostatic terms of FFs may influence partition functions,^80,81^ and also impact the osmotic coefficients of some chemical entities.^82^ Using the paradigmatic system benzamidine/trypsin, we have recently shown that proper parametrization of the ligand torsional potentials is essential to reconstruct the correct ligand binding conformation and free-energy surface.^78^ In particular, the default benzamidine parameters generated by different FFs underestimated the energy of the barrier characterizing the rotation of the amidine group with respect to the aromatic ring. While the numerical value of the ligand/protein binding free energy was not affected by this issue, differences might be found in the energetic profile and binding mechanism obtained using the tailored or default torsional potential. A second important player is the mathematical formulation of molecular charges. Most FFs consider the electrostatic distribution fixed, even though it depends on the phase state, conformation, and the environment surrounding the molecule.^83^ The electron densities of a ligand in the unbound and bound state may also be significantly different.^84^ Although these approximations have a limited impact—in the absolute estimate of electrostatics—on the results quality of MD calculations when taken individually, they introduce errors that can reach tens of kJ/mol, as shown by Kaminsky and Jensen.^85^ Even if such inaccuracies do not affect the detection of the metastable states, the passage through these states, which defines kinetic rates, could be altered. In this regard, Vitalini et al. reported that different FFs might provide contrasting kinetic rates and dynamics for the same system, even when the identified metastable states are in general agreement.^86^ The simplest solution to these issues could be a thorough refinement of the existing FFs to improve the bonded and nonbonded terms. Still, it would not solve the fundamental limit of the classical physics description, especially regarding the molecular electrostatic properties. Instead of lingering on such approaches, we feel that it is time for FFs to step up to a higher tier of modelization, leveraging the computational power of current hardware and more accurate levels of theory. In this sense, the strategy followed by polarizable FFs is particularly promising.

Polarisable FFs adopt a different philosophy when treating molecular electronic densities, allowing the electron cloud to move around the atoms to induce dipole effects and mimic anisotropic electrostatic distributions, such as σ holes. This approach results in a more accurate depiction of molecular charges at the cost of increased computational power.^87^ Several polarizable FFs are available nowadays (e.g., Amber ff02pol, CHARMM Drude, SIBFA, AMOEBA)^88−91^ and they have consistently demonstrated higher capabilities than standard FFs in reproducing thermodynamic data.^86,92,93^ Particularly noteworthy are the results of the SAMPL8 challenge achieved by AMOEBA.^94^ Sadly, these FFs are rarely used to investigate LPB processes, especially due to their high computational cost. They usually find application in studying ion-absorbing, host/guest, or phase transition processes, DNA systems, and protein stability, phenomena where electrostatic interactions play a significant role.^87,95−97^ This is also the case for LPB, so we expect that polarizable FFs will soon become standard for these studies. While polarizable FFs can achieve better results than classic ones, they are still not accurate enough to consistently reproduce kinetic data, as shown by Kaminsky and Jensen.^85^ A further optimization step in FFs development might be the inclusion of kinetic data sets in the FF parameters generation. This proposal is nothing new, seeing as it was already suggested in 2015 by Vitalini et al.^86^ Doing so would, however, require setting up novel parametrization schemes to consider kinetic and thermodynamic measurements. A pragmatic solution to this hurdle could be resorting to ML techniques.

ML has already been employed to optimize existing FFs, like the Lennard-Jones (LJ) parameters for Drude oscillators,^98,99^ or to develop new ones.^100,101^ In particular, LJ parameters are typically computed by taking the experimental hydration-free energy of a compound as a reference. However, their optimization is a lengthy and daunting task requiring extensive validation with multiple experimental data sets. Such an approach is necessary to ensure the reliability and accuracy of a FF. In Chatterjee et al. and Rupakheti et al., a ML approach has been employed to optimize the LJ parameters for several classes of compounds, improving the estimation of different molecular properties, including molecular volume, vaporization and sublimation enthalpies, and dielectric constant.^98,99^ Specifically, Rupakheti et al. reported an improvement in the estimate of molecular volumes and heat of vaporization of the druglike small molecules of 2% and 9%, respectively. Similar improvements were also found in compounds not included in the training set. The average error with respect to the experimental data was 0.46 kcal/mol, which is significantly lower than the average error of 2.0 kcal/mol reported for GAFF using the same compounds.^98^ As previously mentioned, ML is also employed to parametrize new FF from scratch.^100,101^ ML FFs do not employ physics-based equations to describe atom–atom interaction and can be trained using even complex data sets, such as ab initio quantum-mechanical data. The main advantages are that the ML FFs might not require an a priori definition of how the atoms are bonded since the chemical bonds are directly inferred from the pairwise atomic distances, and quantum-mechanics effects can be embedded in the model at significantly reduced computational costs. Several strategies have been implemented to develop ML FFs, with diverse pros and cons and different accuracies. For a more detailed discussion on ML FFs, we refer the reader to the review of Unke et al.^101^ The most notable example of ML FF, as well as one of the most promising, is NequIP from Batzner et al.^102^ Fu et al.^103^ have recently benchmarked it together with other state-of-the-art ML FFs, such as DeepPot-SE,^104^ SchNet,^105^ ForceNet,^106^ and GemNet-T,^107^ using different molecules and observables. In detail, Fu et al. assessed the ML FFs capability to predict the correct forces, distribution of interatomic distances, and sampling of the free-energy surface of organic compounds (ethanol, naphthalene, and salicylic acid), a small molecule (aspirin), and a peptide (alanine dipeptide). They also evaluated whether these ML FFs could reproduce the correct radial distribution functions and diffusion coefficients of condensed phase systems such as liquid water and crystalline superionic lithium. Finally, the numerical stability of each system during the MD calculations was assessed. Overall, NequIP performed consistently better than the other ML FFs, with the exception of the simulation speed (i.e., frames per second). This is due to its construction, since NequIP uses local descriptors that require a higher amount of data to be processed. Notwithstanding, NequIP, as all the other ML FFs, has difficulties reproducing the sampling behavior of standard simulations in systems as simple as alanine dipeptide. In fact, when the simulation of the alanine dipeptide is started from low-density metastable states, NequIP is unable to reproduce the correct probability distribution, resulting in an incorrect free-energy surface. In some cases, simulations starting from these states could also be affected by numerical instabilities and crashes. The authors hypothesize that such issues are due to the quality of data set used to train the model, which has poor statistics of low-density states and lacks high-energy conformations. They remark that the latter could be particularly useful since it could improve the models’ reliability.^103^ Interestingly, ML techniques have also been applied to generalize atomistic FFs at the Coarse-Grained (CG) level with the final aim of reducing the dimensionality of the molecular representation and capturing the most relevant (slow) degrees of freedom of the simulated system.^100,108^ CG models achieve this scope by merging the atomic particles into single entities called “beads” whose energetic interaction is computed through equations inspired by atomistic FFs or classical physics. The final result is reproducing the macroscopic properties of systems that would have required an unfeasible amount of time and resources using all-atom representation, however maintaining a physically sound description of the molecular entities involved.^109−111^ CG FFs have been successfully employed to study mesoscale processes, supramolecular assembly, protein folding, and protein/lipid interactions.^110,112^ In some cases, they were also used to reconstruct kinetic information related to protein–protein association mechanisms,^113−115^ but for a long time, they were not employed to investigate small molecules and, in particular, LPB.^112,116^ The recent works of Dandekar et al.,^117^ Negami et al.,^118^ and Souza et al.^119^ have dramatically changed the status quo of CG FFs. Using different versions of the popular Martini FF, these authors demonstrated its potential in investigating the binding of small molecules to various classes of targets. In particular, Souza et al. employed the recently released third version of Martini FF,^111,119^ which addressed several limitations of the previous ones.^120−122^ Dandekar et al. and Negami et al. were instead able to reconstruct the LPB kinetics with good approximation.^117,118^ Doing so, however, requires estimating the acceleration factor introduced by coarse-graining, which is known to reduce molecular friction and lower energy barriers due to entropy loss.^116^ This is rarely done in practice. Usually, a generic, empirical acceleration factor from three to eight is provided as a correction to the kinetic estimates. These values are speed-up factors roughly estimated by comparing the diffusion coefficients calculated in pure water systems using Martini and atomistic simulations, and not considering the system under investigation.^117,118^ Despite being useful for mesoscale applications, applying CG FFs to LPB problems suffers from limitations mainly correlated to their coarse nature that should be carefully taken into account. In particular, the lower accuracy of CG modeling implies that many details in the ligand/protein interactions may be approximated. Furthermore, the lack of a bijective function between AA and CG models leads to the obvious consequence that different AA models could be mapped to the same CG structure. In all cases, we recommend dutifully validating the kinetic rates obtained via CG MD calculations against experimental data to adequately estimate the acceleration factor introduced by the CG FFs. These aspects and other limitations of classic CG FFs, such as their thermodynamics-based design and configurational-based CG mapping, could be overcome by ML techniques.^100,108,123−125^ While we expect that these FFs, and the Martini in particular, will continue to play a leading role in investigating many phenomena, they could be integrated or replaced by ML-guided CG FFs in the future. As discussed for the atomistic FFs, the potentiality of ML methods in parametrizing, optimizing, and including additional measurables, such as kinetic data sets, could significantly improve the accuracy of FFs and, therefore, cannot be overlooked. CG-ML FFs would also benefit from the more consistent, data-driven approach in deciding how to map atoms into single beads, as opposed to current strategies that only consider the configurational aspect.^124^ In doing so, they could include protein conformational freedom, which plays a determining role in ligand binding processes ruled by induced fit or conformational selection and is currently neglected by CG models.

In conclusion, the current findings indicate that existing atomistic and CG FFs are not accurate enough to reconstruct LPB kinetics properly. For this aim, a higher level of theory and a significant change in the parametrization strategies are required. ML FFs hold promise, but regrettably, they are not yet fully developed for general applications. They are facing essential challenges related to instabilities, representation issues, and limitations in transferability, particularly when applied to larger systems.^103,124,126^ However, their rapid development cycle is encouraging, setting them apart from traditional FFs, with numerous new ML FFs being introduced annually.^100,101,103,108,123,125^ For instance, Fu et al. have provided valuable insights into the limitations of current training methods for ML FFs, which are likely to inspire further advancements in the field.^103^ The atomistic ML FFs hold great promise in achieving quantum mechanics-level accuracy in MD simulations, with the potential to introduce greater flexibility in handling configurational and conformational variations in small molecules, peptides, and proteins. This could include simulating tautomeric shifts or changes in protonation states, which are typically not feasible in MD calculations without dedicated algorithms. However, it is realistic to expect another five-ten years of progress to be necessary for a significant breakthrough. A similar time frame was observed in neural networks for visual pattern recognition, which were first developed in 1988 but only surpassed human accuracy thresholds in 2015.^127^

## 4 Sampling

**Figure 3:**
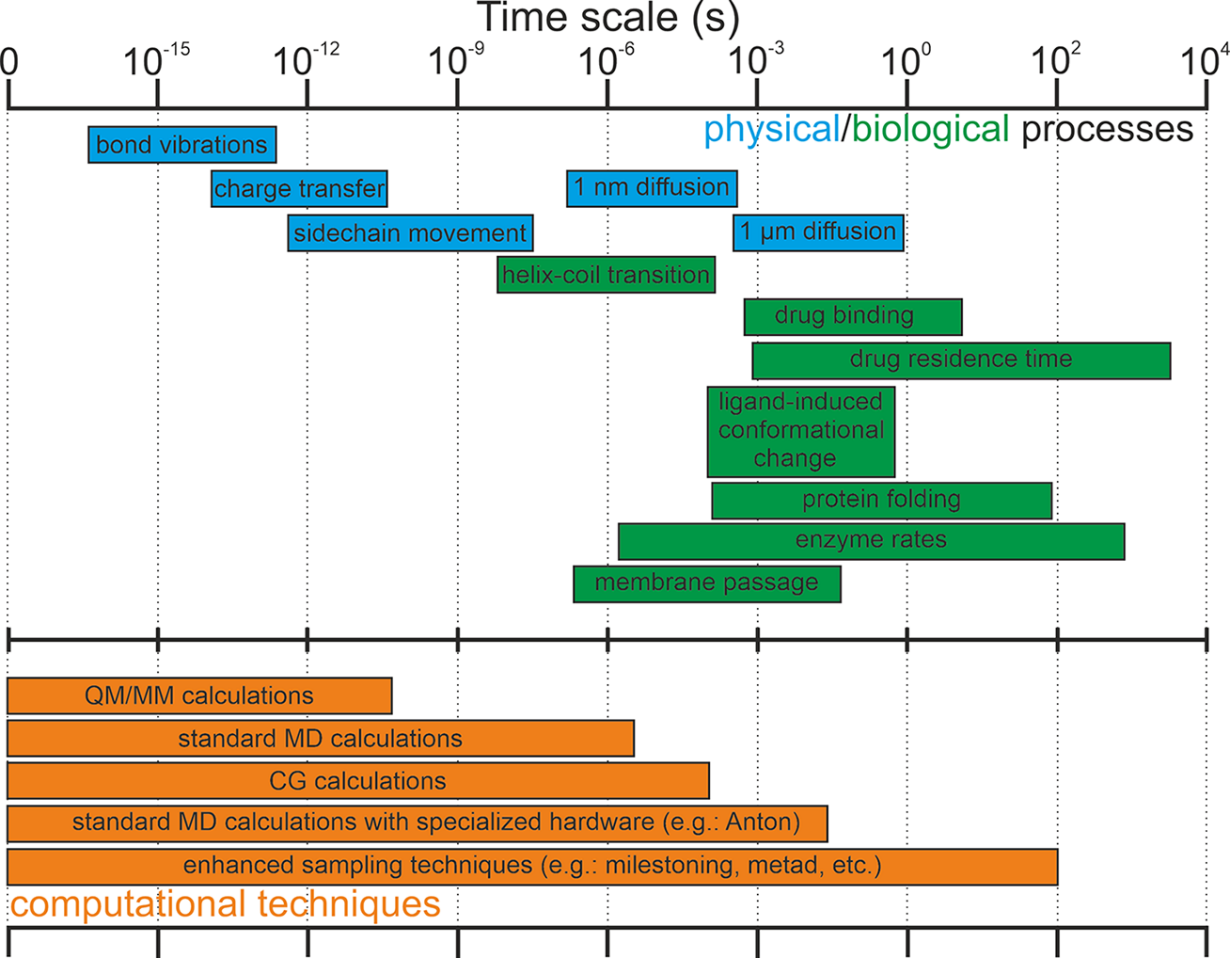
Time scales of the physical and biological processes investigated by simulations. The bars related to the diffusion refer to diffusion coefficients measured in water. At the bottom time scales achievable by computational techniques.

The residence time of a drug in its molecular target can range from milliseconds to hours (Figure 3); therefore, LPB kinetics simulations should reach a comparable time scale.

In addition, to quantitatively estimate the kinetic rates, a relatively large number of transitions between energy minima should be observed to collect meaningful statistics. This makes this kind of study time and computationally demanding.^128,129^ From a theoretical point of view, the study might focus on simulating either the binding or the unbinding, with the estimate of k_*on*_ or k_*off*_, respectively. Once one of the two constants has been computed, the other could be derived according to eq 1 and supposed that K_*b*_ is known.^14^ It has to be said that binding rates are faster than unbinding ones since the unbound state is higher in energy than the bound state, and consequently, the barriers to cross from the unbound state are lower in energy if compared to the same from the bound state. Therefore, the simulation of ligand binding is less time demanding with respect to unbinding. On the other hand, the system in the unbound state has a larger entropy, with multiple isoenergetic conformations possible and only one or a few of them functional for the ligand to reach the final binding mode. This ends in a probabilistic problem that requires a significant number of simulations for sampling the correct ligand binding. At variance with ligand binding, ligand unbinding is characterized by higher energy barriers, but only a few paths can reach the unbound state. This makes the ligand unbinding process and estimate of k_*off*_ computationally less challenging. However, in such simulations, the ligand binding mode should be a priori known. In both cases, kinetic calculations are demanding, and despite the increasing computing power, such extensive simulations are not accessible to many groups. Therefore, the practical solution is to decompose the problem into shorter and simpler tasks or to enhance the sampling.^130−135^ As previously introduced, to estimate kinetic rates and simulate the entire (un)binding process, the transition paths between all of the energetic minimum states have to be sampled. This rules out computational techniques such as end-point and alchemical transformation methods, which focus on calculating the binding thermodynamics based on ligand binding modes, while neglecting the ligand binding mechanism dynamics.^136,137^

The simplest solution to deal with LPB kinetics is to run brute force MD calculations, organized in a number of parallel, independent replicas, followed by an algorithm that computes rates to go from one state to another one.^13^ However, this approach simply shortens the time required to acquire the results without reducing the computational cost. As a result, slow kinetic rates, beyond milliseconds, remain unaccessible to this kind of approach. An elegant solution comes from the concept of a collective variable (CV): a reaction coordinate that distinguishes the diverse states assumed by the system during the binding process. CVs can be either easy and intuitive to define (e.g., geometrical features such as distances between molecules or groups of atoms) or complex in nature (e.g., functions of molecular properties), and they should represent the slowest degrees of freedom of the system under investigation. At the end of the simulation, the potential of mean force (PMF) computed as a CV function describes the evolution of the free energy along the LPB process, allowing the identification of transition states (Figure 1). In real-case scenarios, LPB might be characterized by a relatively large number of degrees of freedom with different dimensions (interatomic distances, angles, coordination shells, etc.), which could be difficult to identify. In addition, ligand binding might be characterized by different phases ruled by diverse slow degrees of freedom, especially in cryptic binding pockets and long kinetics during which the system might significantly evolve through large protein conformational changes, solvation, and other long time scale effects. Considering the complexity of the problem, it is clear that CV definition is a dimensionality reduction problem and user-defined CVs might not be accurate enough.^138−140^ Calculations based on a bad CV choice might lead to simulation issues, including hysteresis and a lack of convergence. In the era of machine learning and semiautomated algorithms, many ways to reduce the dimensionality of the problem have been proposed, including principal component analysis, time-lagged independent component analysis, and various ML approaches.^138,141−148^ These techniques work with a given simulation data set and combine the slowest degrees of freedom of the system using linear or nonlinear functions. In this theoretical framework, the quality of the data set and the time scale of the preparatory simulations heavily affect the results. So far, ML-driven CV definition has been reported on a number of systems and more applications in general cases—where slow degrees of freedom are unknown—are necessary to assess better their predictive power.^13,15,149−152^

Furthermore, it is important to note that the system’s slow degrees of freedom might not necessarily correspond to those functional for LPB procecess. This makes CV selection even more difficult. A possible solution could be to identify the degrees of freedom—and the CVs—functional for LPB (e.g., specific ligand/protein interactions, conformational changes, etc.) by training a ML model with short simulations at relevant metastable states of the system.^153^ A distinct discussion merits the path-based methods, used to analyze the transition between two states by determining a transition-path ensemble constructed from an initial guess.^154−157^ The initial guess pass is enriched by new branches, created, and added to the ensemble by running MD simulations or MC calculations. Despite being elegant in its formulation, this approach can be quite costly for systems with high barriers and rough free-energy profiles, for which possible solutions have been proposed.^158^ To reduce the computational effort, the reaction pathway can be fragmented into discrete states, and the transitions between them can be simulated independently in parallel. This strategy is applied in state-based methods encompassing string methods, Markov state-based models, weighted ensemble, and milestoning, in which the CV can distinguish specific conformations or anchors in Voronoi tesselation.^32,37,38,159−161^ Simulation protocols based on such approaches have been purposely designed for biological systems to consider the presence of multiple metastable states and the typically complex LPB free-energy surface. An interesting, more recent approach is reconstructing the correct sampling probability by operating on enhanced-sampling trajectories with nonoptimized CVs (e.g., variational conformational dynamics, OPES, and similar techniques).^55,162−166^ For example, a customized version of the OPES technique has been applied in the calculation of kinetic rates for the benzamidine/trypsin system,^56^ disclosing the role of water after the definition of a tailored solvation CV by the authors.^57^ Such methods have demonstrated the ability to correct a skewed sampling if the proper degrees of freedom are included; however, as for other similar applications, their predictive power has to be assessed in real cases where little information is known about the system’s slow degrees of freedom.

Whatever method one decides to use to study LPB kinetics and identify TSs, a postprocessing validation step is recommended and good practice. The most intuitive and old procedure implies sampling along nonintersecting hypersurfaces connecting two energy minima A and B identified in previous simulations and computing the committor probability. This is the probability that a given system conformation, belonging to the transition path connecting two minima, ends into A or B.^167−169^ The committor ranges from 0 to 1 and is 0.5 at the TS, which means that running a relatively large number of independent simulations starting from the putative TS, half should fall into A and half into B. It could also be used to identify TSs in simple systems, e.g., with only two energy minima, which is seldom the case in LPB. In addition, statistical tests like the Kolmogorov–Smirnov test might further be used to assess the Markovian transition of the saddle points and the Poissonian distribution of the computed kinetic rates.^13,53,170,171^

Furthermore, committors might be employed to identify optimal reaction coordinates and meaningful CVs, even using ML approaches.^172−174^ This is an active field of research that could be further expanded in the near future.^175^ In conclusion, defining CVs that could at least alleviate the sampling issue is a most reasonable strategy and a daunting task that continues to attract the community.^138,176^ Despite the encouraging results coming from automated and semiautomated ML algorithms, the extensive preparatory simulations required for this kind of calculations limit their applicability.^177^ An interesting direction could be to define the best CVs automatically on-the-fly during simulation using an unsupervised algorithm as suggested by Bhakat.^177^ Independently from the method chosen, the operator is asked to possess a solid understanding of the technique and the investigated system, which dramatically impacts the method’s user-friendliness, as discussed in the next section.

## 5 Friendliness and Performance

In a world more focused on energy efficiency^178,179^ and data openness,^180,181^ aspects such as computing performances, user-friendliness, and code accessibility or reuse are becoming increasingly important. This section briefly discusses the most popular methods for computing the LPB kinetics from this perspective. First, we compare the simulation times required by diverse techniques to compute the association or dissociation rates and the accuracy of their estimates. Then, we discuss code availability, ease of usage, and data openness. We conclude this section by analyzing how the open data approach can be applied to FFs.

Providing a fair comparison of the computational performance of the different techniques is difficult. Nonetheless, some baselines can still be drawn using a common test case like the paradigmatic system benzamidine/trypsin where ligand unbinding occurs with a millisecond time scale.^32,34,39,46,53,57,60,66−68,161,163,182−186^

Table 1 summarizes the results achieved over the years using different methodologies, organized chronologically. Please note that in Table 1, for the sake of discussion, we use the names of the general techniques as defined in the section 2. From the reported data, it can be seen that optimization of the computing protocols, i.e., reduction of computer time needed for LPB simulation, has been a primary focus. From 2016 onward, starting with the work based on the AMS method,^184^ new techniques typically required less than ten microseconds of calculations to provide an estimation of the association and dissociation rates, except for CG MD.^117^ Notable results were achieved by Markovian-WEM (M-WEM)^39^ and OPES_*f*_^57^ in delivering accurate values of K_*off*_ in less than four microseconds. Similarly, MMVT^34,161^ gave an excellent estimate of the K_*on*_ in only five microseconds. The kinetic estimations significantly differed from the experimental ones in all of the other cases. However, one should reckon that such inaccuracy might be due to FFs issues, as discussed in the previous section, and not necessarily to the methods themselves. Considering the continuous improvement in the hardware field^133,187^ and the potentialities of the upcoming quantum computing technology,^188^ it is likely that in the future, the real time needed to achieve the calculation convergence will be significantly shorter. With this in mind, achieving a more accurate, comprehensive description of the interatomic forces (as discussed in section 3) at the cost of more demanding calculations seems to be acceptable or even desirable.

**Table 1:** Comparison of Methods Employed for Computing the LPB Kinetics in the Benzamidine/Trypsin System

Method	*K*_*on*_ (10^6^ M^–1^ s^–1^)	*K*_*off*_ (s^–1^)	Simulation Time^a^ (μs)
Experimental^182^	29	600 ± 300	–
MD + MSM^66^	150 ± 20	95000 ± 33000	50
MD + MSM^67^	440	28000	1
MetaD^53^	11.8 ± 10	9.1 ± 2.5	2
MD + MSM^183^	64	13100 ± 10900	149.1
US + MD + MSM^68^	–b	1170 ± 276.5	58.28
AMS^184^	–b	260 ± 240	2.3
MD + BD^32^	21 ± 3	83 ± 14	19
WEM^185^	–b	5556	4.1
MetaD^163^	–b	4176 ± 324	1.2
WEM^186^	–b	266	8.75
dcTMD^46^	8.7 ± 0.5	270 ± 40	10000^c^
CG MD^117^	368	690000	398
MMVT^34^	120 ± 5	174 ± 9	4.4
LiGaMD^60^	11.5 ± 7.9	3.53 ± 1.41	5
M-WEM^39^	7.6 ± 3.8	769 ± 261	0.73
MMVT^161^	24 ± 2	990 ± 130	5
OPES_*f*_^57^	–b	687^d^	3.3

a Only production runs are considered.

b Data not reported/computed.

c dcTMD employs coarse-graining of the degrees of freedom of the system and increased integration time step (up to 10 fs), significantly reducing the computational time and power needed for simulations^46^

d This value is the slowest unbinding rate identified in 60% of the performed simulations. No estimate of the standard deviation is reported in the paper.^57^

Access to higher computational capabilities or more accurate modeling of molecular properties will be a game changer, but we should not focus on only these aspects. We should also continue to enforce the recent push for good code availability and reusability practices to make these techniques easier to use and comprehend, fostering their diffusion in the scientific community. In principle, computational methods should have clear documentation, self-explaining interfaces, maintained repositories with a tracked list of changes and, when possible, open databases of protocols for reproducibility practices.^180^ For example, MetaD and MMVT algorithms are supported by their specific libraries PLUMED^189^ and SEEKER,^34,161^ with plenty of tutorials and documentation available. Funnel-metadynamics, a variant of metadynamics designed to study LPB, has been recently furnished with a user-friendly protocol, named Funnel-Metadynamics Advanced Protocol (FMAP), and a graphical user interface.^190^ Furthermore, the PLUMED consortium has published PLUMED-NEST, a public repository containing the computational protocols employed in PLUMED-assisted MD calculations, for the sake of data reproducibility.^191^ LiGaMD, on the other hand, has been directly integrated into the Amber software.^192^ We believe that the next step in making this software more accessible and easy to use is integrating it into a web server to generate and validate simulation inputs and create public databases for storing trajectories related to kinetic experiments. Examples of such an approach can already be seen in the CHARMM-GUI Web server,^193^ the 3-dimensional structure Representation Sharing (3dRS),^194^ and the GPCRmd repository,^195^ among others.^196−198^

Lastly, we believe existing and new FFs should be developed following the principles of data openness. Most FFs use diverse definitions for residue names, atom names, or types, which may confuse a novice user. In addition, they have different parametrization routines, which often involve diverse pools of model compounds.^69,73−76,98^ Such discrepancies affect the bonded and nonbonded parameters obtained from the parametrization tasks, leading to divergent behaviors in simulation, especially for small molecules, when diverse FFs are applied to the same problem. Multiple authors have observed such outcomes in different circumstances, in both thermodynamic and kinetic calculations. The SAMPL6 challenge highlighted that GAFF and OPLS systematically overestimated the octanol/water partition coefficients, whereas CGenFF gave more accurate predictions.^81^ Kashefolgheta et al. reported slightly different behaviors of the most popular ligand FFs when evaluating their capability to reproduce experimental cross-solvation free energies.^80^ Zhu showed similar results in its benchmark calculations of ligands FFs when compared to experimental osmotic coefficient data.^82^ Amore et al. demonstrated that different FFs provide varying conformer energies and geometries during the optimization of multiple molecules and fragments.^79^ We documented that the parametrization of benzamidine using default GAFF parameters led to unsatisfactory outcomes, altering the ligand binding conformation and the free-energy landscape of the benzamidine/trypsin binding process.^78^ Kaminsky and Jensen reported that the conformational transitions of various amino acid derivatives varied depending on the FF employed. On average, the errors in the interconversion barriers were in the order of 10 kJ/mol.^85^ While such discrepancies are usually minor and may not be an issue in most cases, they could significantly influence the outcome of LPB kinetic studies, especially when working with poorly parametrized functional groups and atom types,^98,199^ as also shown by the comparison between different versions of OPLS.^74,200,201^ Developing standardized FFs with improved physicochemical description, uniform parametrization protocols, unified validation tests, and reproducible results over a wide array of functional groups would be advantageous for accurately predicting kinetic data and MD calculations.^75^ Creating universal definitions of atom types and names, shared among all FFs, would also help with such standardization efforts. In this context, abandoning the historical classification of atomic entities into atom types, which unnecessarily complicates present FFs due to redundancy issues, in favor of alternative approaches such as the one presented by Mobley et al. could support the development of a gold standard.^202^ A first step in this direction could be the development of public repositories of model compounds with theoretical and experimental data for the parametrization of FFs to avoid discrepancies in the reference data pools. To date, the Open Force Field initiative is the only consortium to host the complete data set employed for the parametrization of its FF in an openly accessible form.^75,76^

## 6 Conclusions

LPB kinetic calculations hold great promise in drug discovery to achieve more accurate prediction models of in vivo drug activity.^10−16^ In the past decade, computational methods have demonstrated the ability to characterize LPB kinetics.^32,34,39,46,53,57,60,66−68,161,163,182−186^ However, the applicability and accuracy of such approaches remain limited (Table 1), mainly due to issues related to FFs and sampling capability. The hardware and software improvements—also considering the emerging era of quantum computing—together with the continuously evolving ML techniques will undoubtedly play a leading role in the coming years, hopefully making this kind of calculations less demanding and useful for ligand database screening protocols. In this regard, one could expect that even quantum mechanical calculations—now considered unfeasible for ligand binding studies^203^—might at least integrate the atomistic level description of LPB. In the present article, we have discussed three macro areas that could be further improved, which are molecular properties parametrization, sampling, and data openness. In addition, to make kinetics calculations the “gold standard” in the near future, the following requisites should also be fulfilled: (i) prediction of kinetic rates; (ii) assessment of the results; (iii) release of the atomistic structure of rate-determining states. To date, kinetics calculations have been employed to reproduce experimental rates, with only a few examples where calculations are presented together with or followed by experimental validation.^26−29^ We expect that a posteriori experimental validation of the simulation data can become a standard practice. This would represent an important acknowledgment of the predictive power of this kind of calculations. Furthermore, the assessment of the results is another crucial point. As introduced in section 4, committor analysis and statistical postprocessing tests are available and should represent a standard practice to assess kinetics constants estimates and identification of TSs. The latter leads to the last point, which is the release of the atomistic structure of the rate-determining states. These are high-energy, short-lived, hence transient, states that are elusive to experimental structural biology techniques like crystallography, Cryo-EM, and NMR. At variance with these techniques, atomistic simulations have the capability of detecting and disclosing the atomistic structure of TSs and these should be reported in any kinetic study. These structures would indeed increase the impact of the work and represent unprecedented structural information helpful for medicinal chemists in the design of ligands with tailored binding kinetic properties. In such a way, the LPB kinetics models could mark a breakthrough in drug discovery and be in the pool of the in silico models as nonanimal alternative methods for drug assessments approved by the FDA (Food and Drug Administration Modernization Act 2.0).^9^
